# Evaluation of the Usability and Acceptability of the InnoWell Platform as Rated by Older Adults: Survey Study

**DOI:** 10.2196/25928

**Published:** 2021-04-21

**Authors:** Haley M LaMonica, Anna E Roberts, Tracey A Davenport, Ian B Hickie

**Affiliations:** 1 Brain and Mind Centre The University of Sydney Camperdown Australia

**Keywords:** older adults, mental health, technology, community-based participatory research, stakeholder participation, smartphone, mobile phone

## Abstract

**Background:**

As the global population ages, there is increased interest in developing strategies to promote health and well-being in later life, thus enabling continued productivity, social engagement, and independence. As older adults use technologies with greater frequency, proficiency, and confidence, health information technologies (HITs) now hold considerable potential as a means to enable broader access to tools and services for the purposes of screening, treatment, monitoring, and ongoing maintenance of health for this group. The InnoWell Platform is a digital tool co-designed with lived experience to facilitate better outcomes by enabling access to a comprehensive multidimensional assessment, the results of which are provided in real time to enable consumers to make informed decisions about clinical and nonclinical care options independently or in collaboration with a health professional.

**Objective:**

This study aims to evaluate the usability and acceptability of a prototype of the InnoWell Platform, co-designed and configured with and for older adults, using self-report surveys.

**Methods:**

Participants were adults 50 years and older who were invited to engage with the InnoWell Platform naturalistically (ie, at their own discretion) for a period of 90 days. In addition, they completed short web-based surveys at baseline regarding their background, health, and mental well-being. After 90 days, participants were asked to complete the System Usability Scale to evaluate the usability and acceptability of the prototyped InnoWell Platform, with the aim of informing the iterative redesign and development of this digital tool before implementation within a health service setting.

**Results:**

A total of 19 participants consented to participate in the study; however, only the data from the 16 participants (mean age 62.8 years, SD 7.5; range 50-72) who completed at least part of the survey at 90 days were included in the analyses. Participants generally reported low levels of psychological distress and good mental well-being. In relation to the InnoWell Platform, the usability scores were suboptimal. Although the InnoWell Platform was noted to be easy to use, participants had difficulty identifying the relevance of the tool for their personal circumstances. Ease of use, the comprehensive nature of the assessment tools, and the ability to track progress over time were favored features of the InnoWell Platform, whereas the need for greater personalization and improved mobile functionality were cited as areas for improvement.

**Conclusions:**

HITs such as the InnoWell Platform have tremendous potential to improve access to cost-effective and low-intensity interventions at scale to improve and maintain mental health and well-being in later life. However, to promote adoption of and continued engagement with such tools, it is essential that these HITs are personalized and relevant for older adult end users, accounting for differences in background, clinical profiles, and levels of need.

## Introduction

### Capitalizing on Technology to Support Health and Well-being

As the global population rapidly ages, there has been an increased focus on the development of strategies to support and maintain health and well-being in later life. As described in detail in our previous work [1], the international literature indicates that approximately two-thirds of adults aged ≥65 years report internet use [2,3], and these older adults also represent the fastest growing group of internet users [4]. Globally, government initiatives have been launched to improve the digital literacy and web-based safety of older adults [5,6]. Thus, using health information technologies (HITs) for mental health screening, intervention delivery, and routine outcome monitoring will be increasingly practical options for older adults.

### The Usability and Acceptability of HITs

Although there are more than 400,000 health care apps available on the market, app use data indicate that most health-related apps have fewer than 10,000 downloads [7]. Recognizing that HITs, such as apps, have enormous potential for empowering self-management [8], the health, medical, and research sectors internationally are prioritizing strategies to enhance community and consumer acceptability, usability, and engagement with such digital tools. Participatory design methodologies facilitate the active participation of key stakeholders in the design of HITs, with the aim of ensuring that the end product meets the needs of the end user, improves usability, and increases engagement of all individuals [9-11]. Despite this evidence, with the exception of a diet diary app for older adults with macular degeneration, few HITs have been designed specifically for older adults.

Importantly, as reported by LaMonica et al [12], the majority of a sample of older adults (198/209, 95%) presenting to a specialized memory clinic reported that they were interested in a web-based tool designed to support healthy aging, including physical health and cognition, self-management of existing conditions, and routine tracking of changes in health outcomes over time. Similarly, most respondents (172/206, 82%) also reported interest in a tool to assess and track mood-related concerns and changes [12]. Given older adults’ interest in and motivation to use HITs to improve health and well-being [12,13], it is critical that HITs are tailored to the older adult community, taking into consideration their unique needs as users.

### The InnoWell Platform

In 2017, the Australian Government Department of Health and InnoWell Pty Ltd (a joint venture between the University of Sydney and PwC [Australia]) entered into a 3-year funding agreement to deliver Project Synergy (2017-2020). The objective of Project Synergy is to conduct a series of collaborative research trials with the specific purpose of co-designing and implementing innovative HITs, including the InnoWell Platform, to enable improved mental health service delivery in Australia, facilitating better outcomes for people with lived experience and their supportive others as well as health professionals and service providers [14]. As detailed in papers by Davenport et al [15] and Iorfino et al [16], the InnoWell Platform comprises a multidimensional assessment targeting a range of biopsychosocial domains to capture a holistic view of the consumer. These data can be complemented by objective behavioral data collected via third-party integrations (eg, Fitbit) and informant-based information, including information provided by supportive others and health professionals. The assessment results are delivered in real time to the consumer at which point they can choose from a range of nonclinical care options (eg, apps and e-tools) that they can engage with immediately. If the consumer is engaged in care through a mental health service, the results are designed to be reviewed collaboratively with a health professional to promote shared decision making in relation to both clinical and nonclinical care options, accounting for consumer preferences. Additional information about the functionality and objectives of the InnoWell Platform is available on the InnoWell website [17].

Through participatory design, we configured a prototype of the InnoWell Platform specifically for older adults, including modification of health domains, informational material, and care options, to ensure relevance and appropriateness for this end user group [1]. This study, a supplement to the original co-design research, aims to evaluate the usability and acceptability of the prototyped older adult configuration of the InnoWell Platform. It is important to note that the InnoWell Platform is indicated for the support of assessment, monitoring, and management of mental ill health and maintenance of well-being; however, as the digital tool is still being validated through a clinical trial [15], recommendations regarding adherence or frequency of use have not been defined [18]. Rather, consumers are free to engage with the InnoWell Platform as it suits their needs.

## Methods

### Participants

Participants were required to be aged ≥50 years, be proficient in English, and complete the required informed consent process. As the study design was naturalistic, there was no predetermined sample size in relation to the number of participants who were able to engage with the prototype. To align with our previous work, we defined *older adults* as aged ≥50 years [1,12], as age 50 years relates to the onset of disorders in later life [19] as well as the identified age range during which it is recommended to address risk factors (ie, cardiovascular disease, obesity, diabetes, etc) known to interfere with healthy aging [20].

This study was advertised through the University of Sydney’s Brain and Mind Centre (BMC) research clinics and private organizations (ie, InnoWell) associated with the BMC. Interested participants were directed to a study-specific webpage on REDCap (Research Electronic Data Capture), a research data collection tool, where they were able to read detailed information about the study before providing consent electronically. Recruitment ran for 5 months (May to September 2020).

### 90-Day Naturalistic Engagement With the InnoWell Platform

Participants were invited to engage with the InnoWell Platform naturalistically (ie, in a manner of their choice) for a period of 90 days; there were no specifications set in terms of frequency or patterns of use. On providing informed consent to participate in the study, the participants received an email invitation to the InnoWell Platform. They were then required to create an account, at which point they were asked to set up their profile by answering a series of demographic questions (ie, year of birth, level of education, and gender at birth). Having established a profile in the InnoWell Platform, participants were asked to complete a comprehensive multidimensional assessment comprising self-report questionnaires assessing a range of biopsychosocial domains specifically tailored to the older adult community (ie, cognition, sleep, and instrumental activities of daily living). The assessment results are then available in real time. In addition, participants were able to access psychoeducational material about all biopsychosocial domains, including clinical care options should that be warranted. In addition, a range of nonclinical care options are available to facilitate the self-management of mental health and well-being. The assessment tools embedded within the InnoWell Platform enable participants to reassess themselves across any or all of the biopsychosocial domains, thus allowing them to track progress over time. Importantly, all steps outlined earlier are voluntary, enabling the participant to discontinue at any time, with the option to return to the InnoWell Platform should they choose to do so. As the InnoWell Platform is designed to be intuitive, enabling independent use by consumers, this approach was believed to best mirror *real-world* engagement with the digital tool, thus facilitating evaluation of the acceptability and usability in this context.

In conjunction with their engagement with the InnoWell Platform, participants were asked to complete short web-based surveys via REDCap at baseline regarding their demographics, health, and well-being, including the Kessler Psychological Distress Scale, an internationally recognized, 10-item scale [21], and the World Health Organization-5 (WHO-5) Well-Being Index, a well-validated, 5-item measure of well-being in older adults [22]. On day 90, participants completed web-based questionnaires about their use of and feedback on the InnoWell Platform as well as the System Usability Scale [23], a 10-item, 5-point Likert-scale evaluating the usability and acceptability of the digital tool. Importantly, no data were collected directly using the InnoWell Platform.

### Data Analysis

Descriptive statistics were used to analyze all aspects of the assessment data. Given that the overall sample size was small (N=16), response options were collapsed for some analyses, combining *strongly agree* and *agree* as well as *strongly disagree* and *disagree*. The Statistical Software Package for Social Sciences version 25 (IBM Corp) was used for all analyses.

### Ethics

The research study was approved by the Human Research Ethics Committee of the University of Sydney (project 2019/172).

## Results

### Demographics

A total of 19 participants consented to participate in the study; however, only the data from the 16 participants (mean age 62.8 years, SD 7.5; range 50-72) who completed at least part of the survey at 90 days were included in the analyses. The demographic information is presented in Table 1. Overall, participants had a minimum of 12 years of education, were married or living with a partner, and were functioning independently without the need for care or support services.

**Table 1:** Participant demographic information.

Demographic and response					Participant, n (%)
**Language**					
	English			16 (100)	
	Other			0 (0)	
**Aboriginal or Torres Strait Islander**					
	No			16 (100)	
	Yes			0 (0)	
**Gender at birth**					
	Female			9 (56)	
	Male			7 (44)	
**Gender identification**					
	Female			9 (56)	
	Male			7 (44)	
**Sexual orientation**					
	Bisexual			1 (6)	
	Gay or lesbian			0 (0)	
	Prefer not to answer			1 (6)	
	Straight			14 (88)	
**Highest level of education**					
	Postgraduate diploma, masters, or PhD			6 (37)	
	Undergraduate degree			5 (31)	
	Certificate or diploma (includes TAFE^a^ and trade qualification)			3 (19)	
	Year 12 or equivalent			2 (13)	
**Relationship status**					
	Divorced			4 (25)	
	Married or living with partner			10 (63)	
	Separated (but still legally married)			1 (6)	
	Single (and have never been married)			1 (6)	
**Living circumstances^b^**					
	Living with family (including partners and dependents), friends, or flat mates			12 (75)	
	Living on my own			3 (19)	
	Living in a retirement village or self-care unit			1 (6)	
**Do you have children?**					
	Yes			12 (75)	
	No			4 (25)	
**Do you have a disability?**					
	Yes			1 (6)	
	No			15 (94)	
**Do you receive a government-based benefit?**					
	**Yes**			6 (38)	
		Age pension	3 (49)		
		Carer allowance	1 (17)		
		Financial assistance for carers (eg, care payment, carer allowance, and carer supplement)	1 (17)		
		Other	1 (17)		
	No			10 (62)	

^a^TAFE: Technical and Further Education.

^b^All participants lived independently.

### Self-Reported Mental Health and Well-being

In relation to mental health and well-being, participants generally reported low levels of psychological distress (median 16.2, range 10-32 with a score of 50 representing the most severe level of psychological distress), although 2 participants indicated high (26) or very high (32) distress levels. Similarly, most participants endorsed good mental well-being (median 64.0, range 12.0-96.0 out of a possible 100 points); however, one participant had a percentage score of 12 on the WHO-5, reflecting the worst possible well-being according to established scoring procedures [24].

### Use of the InnoWell Platform

**Figure 1:**
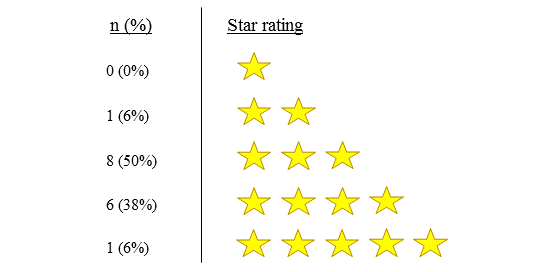
Participant star ratings of the InnoWell Platform (N=16).

Table 2 reflects the frequency and regularity with which participants engaged with the InnoWell Platform, with most participants having used the digital tool only once, at the point of the initial invitation. Participants were unsure (indicating *maybe*) if the InnoWell Platform would be useful for individuals with mental health concerns, and, as shown in Figure 1, they gave it an average rating of 3 stars.

**Table 2:** Patterns of use of the InnoWell Platform.

Question			Participant, n (%)^a^
**When did you first use the InnoWell Platform?**			
	Today	2 (12)	
	Less than a week ago	0 (0)	
	Less than a month ago	0 (0)	
	More than a month ago	4 (25)	
	Approximately 2 months ago	2 (12)	
	Approximately 3 months ago	8 (50)	
**How often did you use the InnoWell Platform?**			
	Every day or almost every day	0 (0)	
	Once or twice a week	0 (0)	
	Once or twice a month	3 (19)	
	Less than once a month	13 (81)	
**How many times (in total) have you logged into the InnoWell Platform?**			
	1 time	8 (53)	
	2-5 times	6 (40)	
	6-10 times	1 (7)	
	11-20 times	0 (0)	
	>20 times	0 (0)	
**When using the InnoWell Platform how long did you normally stay logged on?**			
	1-5 min	7 (47)	
	6-10 min	5 (33)	
	11-20 min	3 (20)	
	21-30 min	0 (0)	
**When would you most commonly use the InnoWell Platform?**			
	Early morning (5 AM to 9 AM)	2 (14)	
	Midmorning (9 AM to noon)	3 (21)	
	Early afternoon (noon to 3 PM)	0 (0)	
	Midafternoon (3 PM to 6 PM)	5 (36)	
	Evening (6 PM to 11 PM)	4 (29)	
	Night time (11 PM to 5 AM)	0 (0)	
**What device did you most commonly use to access the InnoWell Platform?**			
	Personal laptop	6 (38)	
	Smartphone	4 (25)	
	Tablet	3 (19)	
	Personal desktop computer	2 (12)	
	Shared desktop computer	1 (6)	
**Do you think the InnoWell Platform is useful or helpful for people with mental health concerns?**			
	No	0 (0)	
	Maybe	11 (69)	
	Yes	5 (31)	
**Do you like the InnoWell Platform?**			
	No	1 (6)	
	Maybe	9 (56)	
	Yes	6 (38)	
**If it was still available, how many times do you think you might use the InnoWell Platform in the next 12 months?**			
	None	4 (25)	
	1-2 times	3 (19)	
	3-10 times	8 (50)	
	10-50 times	1 (6)	
	>50 times	0 (0)	

^a^In some instances, percentages do not sum to 100 due to rounding errors.

### Usability and Acceptability of the InnoWell Platform

The usability ratings of the InnoWell Platform are summarized in Table 3. Overall, participants reported a suboptimal user experience (median 65, range 45-100 out of a possible 100 points). Although they did not indicate that the InnoWell Platform was difficult to use or overly complex, most respondents noted that they were unsure if they would use the digital tool.

As shown in Textbox 1, participants’ qualitative feedback on their initial impressions of the InnoWell Platform also varied, with one participant describing it as “impressive”, whereas another stated, “I wasn’t sure how to use it.”

Textbox 2 highlights the participants’ favorite features of the InnoWell Platform, including ease of use, the comprehensive nature of the multidimensional assessment, and the ability to track health status over time, all of which are core elements of its conceptualization and design [7,8].

**Figure 2:**
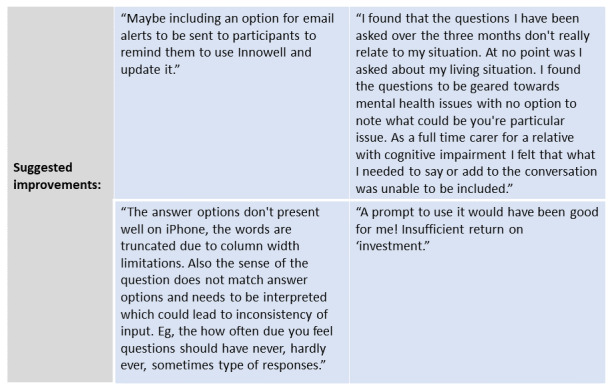
Suggested improvements to the InnoWell Platform.

Importantly, participants also provided valuable suggestions as to how best to improve the InnoWell Platform to enhance the user experience and promote engagement, including the need for greater personalization as well as improved technical functionality to enable use on different devices (Figure 2).

**Table 3:** System Usability Scale ratings of the InnoWell Platform (n=15).

Statement	Participant, n (%)		
	“Strongly disagree” or “disagree”	“Neutral”	“Strongly agree” or “agree”
I think that I would like to use this system frequently.	4 (27)	8 (53)	3 (20)
I found the system unnecessarily complex.	11 (73)	4 (27)	0 (0)
I thought the system was easy to use.	0 (0)	5 (33)	10 (67)
I think that I would need the support of a technical person to be able to use this system.	8 (53)	7 (47)	0 (0)
I found the various functions in this system were well integrated.	0 (0)	10 (67)	5 (33)
I thought there was too much inconsistency in this system.	10 (67)	5 (33)	0 (0)
I would imagine that most people would learn to use this system very quickly.	0 (0)	5 (33)	10 (66)
I found the system very cumbersome to use.	11 (73)	4 (27)	0 (0)
I felt very confident using the system.	0 (0)	7 (47)	8 (53)
I needed to learn a lot of things before I could get going with this system.	8 (53)	6 (40)	1 (7)

**Initial impressions of the InnoWell Platform.**

**What were your first impressions of the InnoWell Platform?**

- Positive
  - “Impressive and if used with your GP (general practitioner) & Psychologist it offers a much better set of tools for managing depression and anxiety than doing the simple DAS (Depression, Anxiety, Stress) scales.”
  - “Easy to use, relevant, accessible. Favourable impression overall”
  - “Quite good”
  - “The ease of use, it was simple and easy to navigate on a mobile phone.”
  - “I thought it was very useful in directing your attention to those aspects of your lifestyle which were likely to affect physical and mental health outcomes”
  - “Clean, easy to use interface”
- Neutral
  - “InnoWell is a program for assessing the mental health of members of the community”
  - “Given that I don’t appear to have any real issues it is hard to comment on the need for reassurance or help”
  - “Program might be of use”
  - “I have to admit I only used it once and now I cannot find it. Maybe I don’t need it right now...”
- Negative
  - “Never even looked at it, until you asked me to evaluate it...”
  - “I wasn’t sure how to use it”
  - “The system assumes user curiosity about negative aspects. Not enough reward built in. Not sure what it was or what value it would be”
  - “It could be that the number of times I felt certain ways could be inaccurate. Also it relies on the honesty of the participant.”

**Preferred features of the InnoWell Platform.**

**What do you like best about the InnoWell Platform?**

- “The ability to chart states of health/well-being over period of time.”
- “Self help resources”
- “That it alerted the user to health considerations.”
- “Quick & easy”
- “It provides a quick assessment of my mental state”
- “It focuses the mind about mental health”
- “Comprehensive range of health areas covered; Opportunity to question/challenge oneself about issues, health in general”
- “It covers a comprehensive range of scales that are integrated and presented simply through the dashboard which can flag issues to discuss with mental health support”
- “Easy to use”
- “It seeks to help and direct if required”
- “I found it beneficial to check in regularly on my mental health”

## Discussion

### Principal Findings

Although older adults are interested in and willing to engage with HITs to support health and well-being [1,12], because of age-related changes in cognition, vision, hearing, and perception as well as health-related needs and risk factors, it is critical that HITs are tailored to the older adult community, accounting for their unique requirements as users. Having engaged with the prototype of the InnoWell Platform for older adults, participants did not report difficulty in using the digital tool, with several describing it as *easy to use;* however, overall usability scores were subpar, potentially because of a lack of relevance to the individuals’ current circumstances and health-related needs (or lack thereof in the case of this generally healthy sample). In other words, a clear purpose for using the InnoWell Platform may have been needed to promote engagement (eg, “Not sure what it was or what value it would be”), a finding that aligns with a previous review of factors that impact acceptance of HITs by older adults [25]. As the participants generally characterized themselves as healthy and independent, experiencing low levels of psychological distress and good mental well-being, they may not have been intrinsically motivated to engage with the InnoWell Platform at this time. It is also plausible that older adults were satisfied with the outcomes of their initial self-assessment and, therefore, did not have a reason to engage further with the InnoWell Platform. The authors of a recent systematic review of HITs for the promotion of well-being of older adults came to a similar conclusion on the limited effects of digital interventions on the mental well-being of older individuals without notable health or social support requirements [26].

Older adults may also be less inclined to use HITs in isolation but rather have a firm desire for such tools to be integrated with standard care practices to enable the therapeutic relationship with health professionals [1]. This may be a particularly important consideration for this consumer group, as they tend to experience greater degrees of social isolation and loneliness [27]. Therefore, the likelihood of adoption of HITs, such as the InnoWell Platform, may be improved if they are recommended by a health professional, a finding that is supported by previous research [28,29]. In light of these results, we aim to develop functionality to better personalize the InnoWell Platform at the individual level to enhance the user experience and to implement and rigorously evaluate the impact of the enhanced digital tool when embedded within health services providing care to older adults as a means to improve outcomes, thus filling an identified gap in the literature [26].

Whether used independently or as part of standard care, HITs are becoming increasingly sophisticated to support healthy aging and prevent disease and disability, thus enabling independent living. Although our participants did not experience difficulty using the InnoWell Platform, a lack of familiarity with or confidence in using technologies has been identified in other studies as a potential barrier to uptake and adoption by older adults [30-32], specifically in relation to web-based health care information seeking [33]. As such, it is important to consider demonstrations and training opportunities for older adults who might otherwise not have the opportunity to learn to use available technologies [34]. This might include videos and instruction guides embedded within the digital tool itself or access to a digital navigator through a clinical service for assistance with technology set up and troubleshooting as needed [35].

### Limitations

This study has some limitations that are important to note. The small sample size may limit the applicability and generalizability of the findings to the general population. In addition, it will be important to further test the perceived usability and acceptability of the digital tool with help-seeking older adults, for whom content and functionality may be more relevant. This study would also have been enhanced by tracking patterns of use and the application of system analytics to better understand how the older adults had engaged with the InnoWell Platform. The use of embedded analytics tools such as Google Analytics should be considered for future evaluation studies to investigate the relationship between participant characteristics and use data. Finally, we did not include a measure of digital literacy, which may have impacted the participants’ feedback on the usability and acceptability of the InnoWell Platform, although little difficulty in using the digital tool was reported by participants.

### Conclusions

It has been demonstrated that older adults will only adopt new technologies when their apparent usefulness and usability outweigh concerns related to technological complexity and decreased social connections [36]. However, reflecting the need to embrace technology as a result of COVID-19 restrictions, a recent survey by the Global Centre for Modern Ageing highlighted that 23% of Australians aged ≥60 years used technology that was previously unfamiliar to them (eg, tablets, apps, and videoconferencing), with 56% of that group indicating that they felt confident in using this new technology [37]. These findings highlight the tremendous opportunity to engage older adults with HITs to support their mental health and well-being, either through direct-to-consumer approaches or as part of standard care. However, this study helps to establish and confirm that it is critical that the design and purpose of any HITs are relevant, appropriate, and personalized for older adult end users, accounting for differing demographic factors, interests, clinical profiles, and levels of need. As demonstrated in this study, the evaluation of HITs helps capture practical feedback on the design of HITs, allowing for iterative refinement before broader implementation, thus facilitating engagement and adoption.

## Abbreviations

BMC: Brain and Mind Centre
HIT: health information technology
REDCap: Research Electronic Data Capture
WHO-5: World Health Organization-5
